# Trans-chalcone and quercetin down-regulate fatty acid synthase gene expression and reduce ergosterol content in the human pathogenic dermatophyte *Trichophyton rubrum*

**DOI:** 10.1186/1472-6882-13-229

**Published:** 2013-09-17

**Authors:** Tamires Aparecida Bitencourt, Tatiana Takahasi Komoto, Bruna Gabriele Massaroto, Carlos Eduardo Saraiva Miranda, Rene Oliveira Beleboni, Mozart Marins, Ana Lúcia Fachin

**Affiliations:** 1 Unidade de Biotecnologia, Universidade de Ribeirão Preto, Av: Costábile Romano 2201, 14096-900, Ribeirão Preto, SP, Brasil

**Keywords:** Antifungal activity, Antifungal agent, Antifungal target, Dermatophytes, *Trichophyton rubrum*

## Abstract

**Background:**

Fatty acid synthase (FAS) is a promising antifungal target due to its marked structural differences between fungal and mammalian cells. The aim of this study was to evaluate the antifungal activity of flavonoids described in the scientific literature as FAS inhibitors (quercetin, trans-chalcone, ellagic acid, luteolin, galangin, and genistein) against the dermatophyte *Trichophyton rubrum* and their effects on fatty acid and ergosterol synthesis.

**Methods:**

The antifungal activity of the natural products was tested by the microdilution assay for determination of the minimum inhibitory concentration (MIC). The effect of the compounds on the cell membrane was evaluated using a protoplast regeneration assay. Ergosterol content was quantified by spectrophotometry. Inhibition of FAS by flavonoids was evaluated by an enzymatic assay to determine IC_50_ values. Quantitative RT-PCR was used to measure transcription levels of the *FAS1* and *ERG6* genes involved in fatty acid and ergosterol biosynthesis, respectively, during exposure of *T. rubrum* to the flavonoids tested.

**Results:**

The flavonoids quercetin and trans-chalcone were effective against *T. rubrum*, with MICs of 125 and 7.5 μg/mL for the wild-type strain (MYA3108) and of 63 and 1.9 μg/mL for the ABC transporter mutant strain (Δ*TruMDR2)*, respectively. The MICs of the fluconazole and cerulenin controls were 63 and 125 μg/mL for the wild-type strain and 30 and 15 μg/mL for the mutant strain, respectively. Quercetin and trans-chalcone also reduced ergosterol content in the two strains, indicating that interference with fatty acid and ergosterol synthesis caused cell membrane disruption. The MIC of quercetin reduced the number of regenerated protoplasts by 30.26% (wild-type strain) and by 91.66% (mutant strain). Half the MIC (0.5 MIC) of quercetin did not reduce the number of regenerated wild-type fungal colonies, but caused a 36.19% reduction in the number of mutant strain protoplasts. In contrast, the MIC and 0.5 MIC of trans-chalcone and cerulenin drastically reduced protoplast regeneration in the two strains. The *FAS1* gene was repressed in the presence of MICs of quercetin, trans-chalcone, fluconazole and cerulenin. The *ERG6* gene was induced in the presence of MICs of fluconazole and cerulenin and was repressed in the presence of MICs of trans-chalcone and quercetin. Trans-chalcone and quercetin inhibited the enzymatic activity of FAS, with IC_50_ values of 68.23 and 17.1 μg/mL, respectively.

**Conclusion:**

Trans-chalcone and quercetin showed antifungal activity against *T. rubrum*, reducing ergosterol levels and modulating the expression of *FAS1* and *ERG6*.

## Background

Dermatophytosis is a cutaneous mycosis caused by fungi of the family Arthrodermataceae (Dermatophytes), which are able to digest keratin. This mycosis is a common infection worldwide [1]. The main etiological agent of dermatophytosis is the anthropophilic and cosmopolitan fungus *Trichophyton rubrum*, which accounts for 69.5% of all dermatophytic infections [2,3]. Infections caused by this species are difficult to treat and there is only a limited number of antifungal drugs available for clinical use, especially when compared to the arsenal of antibacterial drugs [4,5].

Therefore, novel drugs with more specific and effective mechanisms of action against dermatophytes are urgently needed. In this respect, natural products provide a rich source of chemical diversity for the development of new drugs [6]. However, the lack of suitable targets and the similarities between fungal and mammalian cells often result in compounds that are highly toxic to humans [7,8]. An interesting target is fatty acid synthase (FAS), an enzyme that participates in endogenous fatty acid synthesis [9]. The fact that this enzyme shows marked structural differences between fungal and mammalian cells makes it a promising target for the development of new antifungal drugs [10]. The best known natural inhibitor of FAS is cerulenin, an epoxide produced by the fungus *Cephalosporium caeruleus*. This compound covalently binds to the catalytic site of FAS and disrupts the condensation reaction of acetyl-COA and malonyl-COA, inhibiting the biosynthesis of fatty acids and sterols in yeast [11].

In this study, we explored the antifungal activity of quercetin and five other flavonoids described as inhibitors of FAS against *T. rubrum*[12]. DNA microarray studies using *T. rubrum* and synthetic inhibitors of FAS (PHS11A and PHS11B) have shown marked transcriptional modulation of several genes such as *FAS1* and *FAS2*, *ERG6* (a gene involved in ergosterol metabolism), and multidrug resistance genes [13,14]. We evaluated the antifungal activity of flavonoids against *T. rubrum* and their effects on fatty acid and ergosterol synthesis using a wild-type strain and a mutant strain (Δ*TruMDR2*) that carries a disrupted version of an ABC transporter involved in multidrug resistance [15].

## Methods

### Fungal strains

The *T. rubrum* strain H6 (ATCC MYA3108) and the mutant strain Δ*TruMDR2* (obtained by disruption of the *TruMDR2* gene of strain MYA3108) were submitted to standard techniques for fungal manipulation and growth as described previously by Fachin et al. [15].

### Natural products and chemicals

**Figure 1:**
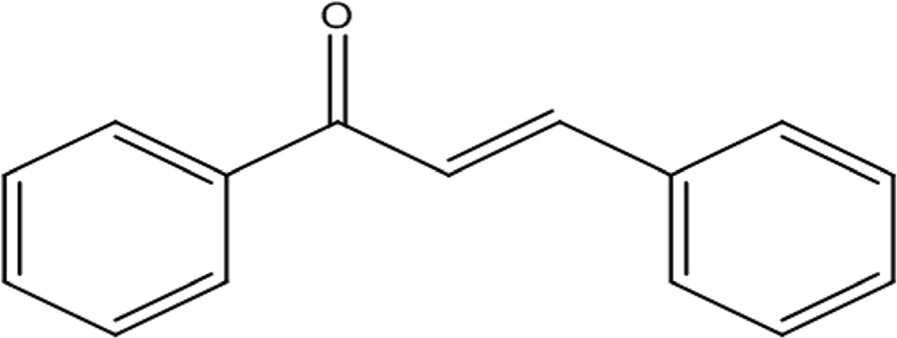
Non-substituted chalcone (Trans-chalcone).

Cerulenin and the natural products were purchased from Sigma-Aldrich (St. Louis, MO, USA). Quercetin and cerulenin were diluted in 10% aqueous DMSO solution and trans-chalcone (Figure 1) was diluted in 5% aqueous DMSO. Ellagic acid and galangin were diluted in 20% ethanol, and luteolin and genistein were diluted in 10% methanol. Fluconazole was provided by the Pharmacy of the University of Ribeirão Preto and was diluted in 5% aqueous DMSO. The final concentration of all solvents (DMSO, ethanol, and methanol) used in the antifungal assay was fixed at a maximum of 0.5%. Fluconazole and cerulenin were used as positive controls. The solvent controls consisted of the solvent (DMSO, ethanol, or methanol) without the tested flavonoids at a final concentration of 0.5%.

### Antifungal assay

Susceptibility of the MYA3108 and Δ*TruMDR2 T. rubrum* strains (1.0 to 8 × 10^5^ CFU/mL) was tested by determining the minimum inhibitory concentration (MIC) of different concentrations of the flavonoids (0.475 to 1000 μg/mL) and controls (fluconazole and cerulenin: 1.9 to 1000 μg/mL) using the M38-A microdilution technique proposed by the Clinical and Laboratory Standards Institute (CLSI, 2002) and described by Fachin et al. [15]. Microtiter trays were incubated at 28°C and MICs were recorded after 7 days of incubation. The MIC_100_ was defined as the lowest concentration of flavonoids that completely inhibited the growth of MYA3108 and Δ*TruMDR2*. The assays were carried out in three independent experiments performed in triplicate.

### Quantification of ergosterol content

Mycelia of strain MYA3108 or Δ*TruMDR2* were incubated in 50 mL liquid Sabouraud glucose medium for 24 h at 28°C under gentle shaking at 200 rpm. The mycelia were harvested aseptically and transferred to 20 mL fresh Sabouraud medium containing MICs of quercetin and of the controls cerulenin and fluconazole. The MIC and 0.5 MIC were used for trans-chalcone. Next, the material was incubated for 48 h under the same conditions as described above and harvested by filtration. Ergosterol was extracted as described by Arthington-Skaggs et al. (1999) [16] and quantified spectrophotometrically based on a standard curve of different concentrations of ergosterol (Sigma).

### Protoplast regeneration assay

Protoplasts were obtained from each *T. rubrum* strain grown for 7 days in 30 mL lytic solution (20 mg/mL *Trichoderma harzianum* lysing enzymes purchased from Sigma-Aldrich; 0.7 M KCl and 1 M MgSO_4_, pH 6.8) by incubating the mycelium for approximately 4–6 h at 28°C under gentle agitation. The protoplasts were filtered through glass wool, collected by centrifugation (5 min, 4°C, 1556 *g*), and washed in 10 mL regeneration buffer (0.8 M NaCl, 10 mM CaCl_2_, 50 mM Tris–HCl, pH 7.5). The concentration of cells was adjusted to 1 × 10^4^ protoplasts/mL. Regenerated protoplasts were selected on solid minimal medium [17] supplemented with 1 M sucrose, 0.2% casein and 0.07 mM NaNO_3_ by incubation with the MIC and 0.5 MIC concentrations of quercetin, trans-chalcone and cerulenin for 7 days at 28°C.

### Quantitative RT-PCR

Quantitative RT-PCR was used to evaluate the transcription level of the *FAS1* and *ERG6* genes involved in fatty acid and ergosterol biosynthesis, respectively, during exposure of *T. rubrum* to the flavonoids. Total RNA was extracted from approximately 30 mg mycelia grown for 16 h in the presence of MICs of quercetin, trans-chalcone, cerulenin, and fluconazole as described by Fachin et al. [15] using the Ilustra RNAspin Mini RNA Isolation kit (GE Healthcare). Mycelia grown in the presence of 1.25% DMSO (Sigma) were used as control. Complementary DNA was synthesized from 2 μg total RNA in a 20-μL reaction volume using the RevertAID H Minus First Strand cDNA Synthesis kit (Fermentas^®^). The quantitative RT-PCR experiments were performed in triplicate using the SYBR Taq Ready Mix kit (Sigma) on an Mx3300 QPCR system (Stratagene). The gene-specific primers are shown in Table 1. Expression levels were calculated by the comparative Ct method using 18S rRNA as normalizer gene and untreated mycelia as reference. The results are reported as the mean ± standard deviation of three experiments.

**Table 1:** Primers used for RT-PCR

**Gene**	**Sequence**	**Size (bp)**	**Reference**
18S	F: 5’CGCTGGCTTCTTAGAGGGACTAT-3’	51	[18]
	R: 5’-TGCCTCAAACTTCCATCGACTT-3’		
*FAS1*	F: 5’-CGAACTGCTGAAAGTGGCG-3’	54	[18]
	R:5’-TGGGTGGTAGGTGAAGTAGAACG-3’		
*ERG6*	F: 5’-CTCTGGCAAGACACGAACAC-3’	126	This paper
	R:’5’-CCTTGCAGCCGGTGAAGG-3’		

### Extraction of total proteins for enzymatic assay

The method described by Li et al. [12] was used for the extraction of total proteins, with modifications. Total proteins were extracted from the yeast *Saccharomyces cerevisiae* using glass beads and 20 mg/mL of lysing enzymes (Sigma) in 125 mM phosphate buffer, pH 6.6, 1 mM EDTA, 1 mM DTT, 0.7 μg/mL pepstatin, 0.2 μg/mL aprotinin, and 0.2 μg/mL leupeptin. The lysate was centrifuged at 30,000 *g* for 30 min at 4°C and precipitated with 25% ammonium sulfate for 30 min under shaking on ice. The protein pellet was obtained by centrifugation at 12,000 *g* for 30 min at 4°C. The extracted proteins were resuspended in the same buffer and dialyzed for 24 h in phosphate buffer. Total proteins were quantified and then stored at 4-8°C for one week.

### FAS enzymatic assay

The enzymatic assay was performed according to Li et al. [12], with modifications. Total proteins were quantified and diluted (500 mOD/min). The assay of FAS inhibition by flavonoids was carried out in 96-well plates in a final volume of 100 μL. First, the previously diluted proteins were incubated with quercetin and trans-chalcone for 30 min at room temperature. Next, 50 μL of this mixture was added to 250 mM reaction phosphate buffer containing 1 mM malonyl CoA, 1 mM NADPH, and 40 μM acetyl CoA. The microplate was read immediately for 10 min at 340 nm. The readings were obtained at intervals of 2 min until the end of analysis. FAS activity was calculated by subtracting the OD value obtained after 1 min from the OD value obtained after 10 min. The remaining activity was obtained in the same way and inhibition was calculated by comparison between control activity (considered to be 100%) and remaining activities of the treatments. The IC_50_ was determined by linear regression. The negative control consisted of protein plus reaction buffer. DMSO (1%) was also tested and did not interfere with FAS activity. Another control consisted of buffer reaction without protein.

### Statistical analysis

Means were compared by the Scott-Knott test and a *P* value <0.05 was considered to indicate statistically significant differences.

## Results

### Antifungal assay

Quercetin and trans-chalcone were the most effective compounds against both the wild-type (MYA3108) and mutant strain (ΔTruMDR2) of *T. rubrum*. In contrast, no antifungal activity was observed for ellagic acid, galangin or genistein. Luteolin exhibited antifungal activity against both strains, but to a lesser extent than trans-chalcone and quercetin against the wild-type *T. rubrum* strain. The MIC of quercetin was 125 μg/mL for strain MYA3108 and 63 μg/mL for Δ*TruMDR2*. Similar MICs were obtained for cerulenin, which was used as a positive control. However, the highest inhibitory activity against the *T. rubrum* strains was observed for trans-chalcone, which exhibited markedly lower MICs (MYA3108: 7.5 μg/mL, Δ*TruMDR2*: 1.9 μg/mL) than the positive controls fluconazole and cerulenin (Table 2). No antifungal activity was observed for any of the solvent controls at the final concentration tested (0.5%).

**Table 2:** **MIC**_**100 **_**(μg/mL) of the natural products against *Trichophyton rubrum***

**Strain**	**CRL**	**FLC**	**QCT**	**GEN**	**EAC**	**LUT**	**GAL**	**T-CHL**
MYA3108	125	63	125	>1000	>1000	250	>1000	7.5
Δ*TruMDR2*	15	30	63	>1000	>1000	63	>1000	1.9

*CRL*: cerulenin; *EAC*: ellagic acid; *FLC*: fluconazole; *GAL*: galangin; *GEN*: genistein; *QCT*: quercetin; *LUT*: luteolin; *T-CHL*: trans-chalcone.

### Quantification of ergosterol content

A reduction in ergosterol content of 33.54% (wild type) and 56.15% (mutant) was observed for the two *T. rubrum* strains cultured in the presence of the MIC of quercetin when compared to untreated cells. This result is comparable to the reduction caused by MICs of fluconazole and cerulenin, which ranged from 40 to 61.5%. In contrast, the 0.5 MIC of trans-chalcone caused a more marked reduction in ergosterol content of 74 to 77% in the two strains. Ergosterol content was reduced by 100% in the presence of the MIC of trans-chalone (Table 3).

**Table 3:** **Percent reduction of ergosterol content in the *Trichophyton rubrum *strains**

**Concentration (μg/mL)**	**Strain MYA3108**	***ΔTruMDR2***
MIC of quercetin	33.54 ± 8.95^a^	56.15 ± 4.06 ^a^
MIC of cerulenin	40.72 ± 5.6^a^	49.48 ± 8.24 ^a^
MIC of fluconazole	40.95 ± 9.05^a^	61.50 ± 6.73^a^
MIC of trans-chalcone	100 ^b^	100 ^b^
0.5 MIC of trans-chalcone	74.59 ± 5.35 ^c^	77.85 ± 15.73 ^c^

*MIC*: minimum inhibitory concentration.

^a,^^b,^^c^: Values followed by different superscript letters differ significantly between groups.

### Protoplast regeneration assay

As can be seen in Table 4, trans-chalcone was the best compound to inhibit protoplast regeneration in the two *T. rubrum* strains when tested at the MIC and 0.5 MIC. Similar results were obtained for cerulenin, a finding suggesting that trans-chalcone also acts by disrupting cell membrane homeostasis. Interestingly, protoplast regeneration was higher in the mutant strain than in the wild-type strain in the presence of 0.5 MIC of trans-chalcone. The MIC of quercetin reduced the number of regenerated protoplasts by 30.26% (wild-type strain) and 91.66% (mutant strain). Half the MIC of quercetin did not reduce the number of regenerated wild-type fungal colonies, but caused a 36.19% reduction in the number of mutant strain protoplasts.

**Table 4:** **Percent reduction in the number of regenerated protoplasts in the *Trichophyton rubrum *strains**

**Compound (μg/mL)**	**Strain**	
	MYA3108	Δ*TruMDR2*
MIC of quercetin	30.26 ± 5.78 ^b^	91.66 ± 8.33^d^
MIC of cerulenin	100^c^	100^e^
MIC of trans-chalcone	100^c^	100^e^
0.5 MIC of quercetin	0^a^	36.19 ± 6.44^a^
0.5 MIC of cerulenin	100^c^	65.26 ± 7.61^b^
0.5 MIC of trans-chalcone	100^c^	83.24 ± 3.50^c^

*MIC*: minimum inhibitory concentration.

^a,^^b,^^c,^^d^: Values followed by different superscript letters differ significantly between groups.

### Modulation of *ERG6* and *FAS1*

**Figure 2:**
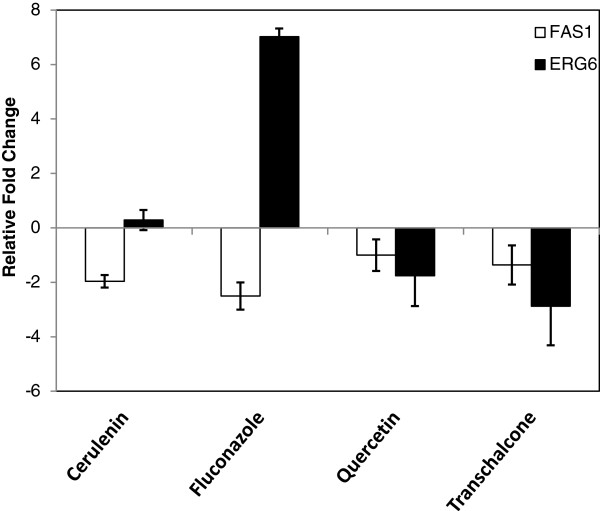
**Relative fold change in the *FAS1 *and *ERG6 *genes determined by quantitative RT-PCR.**

Quantitative RT-PCR was used to evaluate the modulation of *FAS1* and *ERG6* gene expression in the presence of flavonoids. The *FAS1* gene of *T. rubrum* was repressed in the presence of MICs of quercetin, trans-chalcone, fluconazole, and cerulenin. The *ERG6* gene was induced in the presence of MICs of fluconazole and cerulenin and was repressed in the presence of MICs of trans-chalcone and quercetin after 16 h of growth of *T. rubrum* (Figure 2).

### FAS enzymatic assay

The IC_50_ value was 17.1 μg/mL for quercetin and 68.23 μg/mL for trans-chalcone, indicating that quercetin exhibits a better FAS inhibitory activity than trans-chalcone (Table 5).

**Table 5:** Fatty acid synthase inhibitory activity

**Compound**	**IC**_**50 **_**(μg/mL)**
Quercetin	17.1
Trans-chalcone	68.53

## Discussion

Chalcones (1,3-diaryl-2-propen-1-one) are open-chain flavonoids that are mainly synthesized by plants. These compounds have been shown to exert significant antifungal activity, especially against dermatophytes [18]. Most chalcones inhibit the biosynthesis of the yeast cell wall [19], but there is evidence that chalcones also block FAS in yeast (12) and fatty acid synthase II in mycobacteria [20].

In the present study, the MICs of quercetin, trans-chalcone, cerulenin and fluconazole were always lower for the mutant strain than for the wild-type strain (MYA3108). This higher susceptibility of the mutant strain might be explained by disruption of the *TruMDR2* gene. This gene encodes an ABC transporter that is involved in the resistance to various antifungal agents, such as terbinafine, 4NQO, and ethidium bromide [15]. To our knowledge, this is the first study reporting the involvement of the *TruMDR2* transporter in flavonoid-mediated FAS inhibition.

A reduction of ergosterol content was observed in the two *T. rubrum* strains when the fungus was grown in the presence of the MIC of quercetin. However, a higher percent reduction of ergosterol content was found when the strains were grown in the presence of 0.5 MIC and MIC of trans-chalcone, demonstrating a relationship between fatty acid synthesis and ergosterol. In fungi, sterols are found in the plasma membrane where they play a role in membrane permeability.

The protoplast regeneration assay has been used to evaluate the level of plasma membrane damage caused by antifungal compounds [21,22]. Quercetin and trans-chalcone reduced the number of regenerated protoplasts in the two strains, suggesting an effect of these compounds on the cell membrane. Interestingly, in the presence of the 0.5 MICs of cerulenin and trans-chalcone, protoplast regeneration was higher for the mutant strain grown in regeneration medium with a high sucrose concentration (1.5 M) when compared to the wild-type strain. On the other hand, ergosterol content was lower in the mutant strain (11.2 μg/mL) than in the wild-type strain (31.39 μg/mL) when the cells were cultured in Sabouraud medium (data not shown). In yeast, osmotic stress (NaCl and sorbitol) has been shown to increase the level of resistance of cells treated with drugs that inhibit ergosterol synthesis [23]. This phenomenon observed for yeast may explain the better regeneration seen in the *T. rubrum* mutant strain. However, further studies are needed to clarify these results.

The expression of *T. rubrum FAS1* (fatty acid synthases) and *ERG 6* (ergosterol synthesis) genes is modulated by the synthetic FAS inhibitors PHS11A [13] and PHS11B [14]. In this study, we also observed that the inhibition of fatty acid and ergosterol synthesis by quercetin and trans-chalcone may involve transcriptional modulation of these genes since they are down-regulated by both flavonoids*.* In addition to transcriptional modulation of the *FAS1* gene, experiments using *S. cerevisiae* cells have shown that quercetin and trans-chalcone inhibit the enzymatic activity of FAS. Quercetin showed a better inhibitory activity, but trans-chalcone showed a better antifungal activity. This result suggests that the antifungal activity of trans-chalcone may be related to targets other than FAS, such as ergosterol synthesis. Ergosterol is a vital component of the fungal cell, but little is known about the genetics and biochemistry of the ergosterol biosynthesis pathway and only 20 genes involved in the biosynthesis of ergosterol in *T. rubrum* have been sequenced [24]. Zhang et al. [13] demonstrated up-regulation of the *FAS1*, *FAS2* and *ERG6* genes in response to PHS11A. In addition, the *ERG5*, *ERG6* and *ERG25* genes have been shown to be up-regulated in *Candida albicans* strain SC5314-AR, which is resistant to fluconazole and amphotericin B. The higher expression of these genes may increase the conversion of lanosterol to eburicol and 14-methyl fecosterol. By altering the pathway at this particular point, the cell would no longer be susceptible to the effects of fluconazole or amphotericin B. Analysis of sterol content in this strain confirmed the hypothesis that resistance to the two antifungal agents was due to the accumulation of sterol intermediates, which is consistent with the inactivation of lanosterol demethylase, and to the increased expression of several ergosterol biosynthesis genes [25]. According to Leber et al. [26], when ergosterol synthesis is inhibited, steryl esters are hydrolyzed and ergosterol levels remain sufficiently high to maintain membrane formation and growth for some time, and no genes involved in ergosterol synthesis are induced. Furthermore, ergosterol has been reported to regulate its own synthesis by negative feedback mechanisms [27-30].

The complete assessment of the mode of action of quercetin and trans-chalcone will require the analysis of additional genes and other assays that could demonstrate the interference of FAS inhibition with fatty acid homeostasis in *T. rubrum*. One possibility is supplementation of the growth medium with exogenous fatty acids in the presence of quercetin and trans-chalcone. Nevertheless, the present results indicate that these compounds can be explored for the development of new antifungal drugs.

## Conclusion

Quercetin and trans-chalcone presented the best antifungal activity among the flavonoids tested. The results of the FAS enzymatic assay showed that quercetin is a better inhibitor of this enzyme than trans-chalcone. However, the lower MIC of trans-chalcone when compared to quercetin suggests the involvement of another cellular target in addition to FAS. Therefore, trans-chalcone is a potential candidate for the development of new antifungal drugs against *T. rubrum* since it simultaneously inhibits the synthesis of fatty acids and ergosterol, a fact reducing the risk of resistance. This property is another advantage since these two targets are specific of fungal cells.

## Competing interests

The authors declare that they have no competing interests.

## Authors’ contributions

TAB carried out the study; TTK and BGM participated in the evaluation of MIC, ergosterol content and protoplast regeneration assay; CESM supervised the ergosterol content assay; ALF and MM designed the experiments and wrote the manuscript; ROB wrote the manuscript and supervised the work. All authors read and approved the final manuscript.

## Pre-publication history

The pre-publication history for this paper can be accessed here:

http://www.biomedcentral.com/1472-6882/13/229/prepub
